# Can lifestyle preferences help explain the persistent gender gap in academia? The “mothers work less” hypothesis supported for German but not for U.S. early career researchers

**DOI:** 10.1371/journal.pone.0202728

**Published:** 2018-08-28

**Authors:** Monika Sieverding, Constanze Eib, Andreas B. Neubauer, Thomas Stahl

**Affiliations:** 1 Department of Psychology, Heidelberg University, Heidelberg, Germany; 2 Department of Psychology, Uppsala University, Uppsala, Sweden; 3 German Institute for International Educational Research (DIPF), Frankfurt am Main, Germany; 4 Center for Research on Individual Development and Adaptive Education of Children at Risk (IDeA), Frankfurt am Main, Germany; Indiana University Bloomington, UNITED STATES

## Abstract

Do lifestyle preferences contribute to the remaining gender gap in higher positions in academia with highly qualified women—especially those with children—deliberately working fewer hours than men do? We tested the “mothers work less” hypothesis in two samples of early career researchers employed at universities in Germany (*N* = 202) and in the US (*N* = 197). Early career researchers in the US worked on average 6.3 hours more per week than researchers in Germany. In Germany, female early career researchers with children had drastically reduced work hours (around 8 hours per week) compared to male researchers with children and compared to female researchers without children, whereas we found no such effect for U.S. researchers. In addition, we asked how long respondents would ideally want to work (ideal work hours), and results revealed similar effects for ideal work hours. Results support the “mothers work less” hypothesis for German but not for U.S. early career researchers.

## Introduction

The striking gender gap in academic careers is a global phenomenon [1] and is manifested especially after earning the PhD degree: in the United States and Europe, around half of those who obtain doctoral degrees are female, but only every third full professor in the US is female (31% in 2013) [2]. In Germany, this number is even smaller where only every fifth full professor (22% in 2014) is a woman [3]. Earlier research identified biases or discrimination against women in science (see for example [4, 5]). However, a recent extensive review of 20 years of research on discrimination processes in the domains of publishing, funding, and hiring in math-intensive sciences concluded that, at present, these processes could no longer explain the underrepresentation of women in higher positions in science [6, 7]. Instead of gender discrimination, the authors suggested that lifestyle choices and career preferences of women might better explain the remaining gender gap in academia [8].

In this research, we compared the work hours of early career researchers in Germany and the US as a function of country, gender, and parenthood. It is often contended that someone who wants to have a successful career in academia has to work especially long hours [9]. Research productivity, a crucial aspect for a career in academia, is predicted by work hours, such that people working more than 60 hours per week show a sharp increase in productivity over those who work 50 to 59 hours per week [10]. The number of hours worked has been identified as a relevant career predictor [11] and can be regarded as a conscious lifestyle preference and career investment. We decided to use the term preference over choice. Whereas the term ‘choice’ implies the decision between two or more alternatives (family / children OR academic career), the term ‘preferences’ suggests to have different priorities regarding different life areas which result in certain allocations of resources and time. We define ‘lifestyle preferences’ as the individual decision to work less after having a child. With this, we follow arguments and results by Ceci and Williams [6] and Ceci et al. [12] that, nowadays, lifestyle preferences play a more important role in explaining the gender gap in academia compared to gender discrimination processes. For example, a longitudinal study with mathematically precocious males and females showed that highly qualified women differed substantially in their lifestyle preferences from highly qualified men [13]. Ceci and Williams rightly point out that lifestyle preferences are not always freely chosen by the individual: “We conclude that differential gendered outcomes in the real world result from differences in resources attributable to choices, whether free or constrained”([6], p. 3157).

We chose to study early career researchers for two reasons. First, the proportion of women decreases at higher steps of the academic career ladder, which has been called the “leaky pipeline” phenomenon [14, 6] or “falling off the academic bandwagon” [15]. Second, when young researchers stay in academia after earning their PhD degree, they tend to have aspirations to pursue an academic career [16], which might lead to a prestigious senior position in academia such as a full professorship. Therefore, the early career stage is a qualification phase in which work hours seem to be particularly relevant.

We decided to compare the situations of early career researchers in two countries with strong research universities: the US and Germany. Across various professions, reports have suggested a striking difference in hours worked between U.S. and German employees, with U.S. employees experiencing greater pressure to put in long hours [17, 18]. A new report calculated that Europeans’ work time is 19% shorter than that of U.S. citizens [19]. In the case of academia, researchers from the Americas (US, Canada, Brazil) work at least 10 hours longer than German researchers [20]. We therefore predicted that early career researchers working in the US would report longer work hours than early career researchers working in Germany.

The main goal of our research was to explore work hours as a function of gender and parenthood. Prior research has demonstrated that highly qualified women work less than comparable men do. A longitudinal study of 5,000 intellectually talented participants from the US found that highly talented and qualified women (with cognitive abilities at age 13 in the highest 1% of their age group) worked fewer hours and also wanted to work less than comparably talented and qualified men 20 years later, at the age of 33 [8]. The authors posited that gender differences in work hours could contribute to gender differences in vocational success [21]. David Lubinski, who co-directed this longitudinal research, made the point: “One only needs to imagine the ticking of a tenure clock and the differences likely to accrue over a 5-year interval between two faculty members working 45-versus 65-hr weeks (other things being equal)” ([22], p. 106). As prior research from the US found similar patterns in academia with male full-time faculty putting in more work hours than their female counterparts [10, 23], gender differences in work hours may contribute to gender differences in academic success. Therefore, we expected that female early career researchers would be found to work less than male researchers. In addition, similar to Lubinski and colleagues [22, 21], we also investigated how many hours early career researchers ideally want to work (ideal work hours), and we expected a similar gender effect.

More specifically, we predicted that gender differences in (actual and ideal) self-reported work hours would primarily result from the different responsibilities of men and women with regard to (caring for) children. Therefore, we test the idea that children disproportionally affect the work hours of female academics in contrast to male academics, a hypothesis we termed “*mothers work less*”. This hypothesis is based on evidence that for highly qualified women who have successfully finished their PhD, it is still more difficult to balance family and career responsibilities compared with equally qualified men [20, 24]. A postdoc survey conducted in the US in 1999 found that female early career researchers who had children worked less than women without children and men with children [25]. However, we expected differences in the effects of parenthood on working hours between the countries because the parental leave policies are fundamentally different. U.S. mothers can get only up to 12 *weeks* of unpaid leave for each newborn child, whereas German parents—mothers as well as fathers—can get paid leave for up to 14 *months*. Two longitudinal studies of highly educated professionals in Germany found that parenthood had a strong negative influence on women’s career commitment—operationalized by work hours [26] and career interruptions [27]—and subsequently on objective career success. For men, however, there was no such impact of parenthood on work hours, career interruptions, or career success. A recent study comparing the work hours of representative samples of middle-aged adults found a higher prevalence of the male breadwinner model in Germany than in the US [17]. Thus, our prediction was that the postulated Gender x Parenthood interaction would be attenuated among early career researchers in the US compared with Germany.

In addition to gender and parenthood, we controlled for a number of factors that may affect work hours. Specifically, we controlled for: work as a calling [28, 17], mother-child ideology [29, 30], and duration of and years since PhD training. A person with a calling works for the fulfilment the job brings into their life [31]. Individuals who view their work as a calling tend to be motivated to work longer hours and experience their work as meaningful [28]. Another variable that could impact work hours may be gender role attitudes, such that female researchers who endorse a traditional mother-child ideology tend to work fewer hours [29]. For instance, relatively more individuals in Germany (vs. the US) believe that it is harmful to a child if a mother works [30, 32]. Third, we controlled for duration of and years since PhD training, as these variables may be indicators of level of aspiration.

## Materials and methods

### Participants

Participants were invited to fill in an online survey in Germany and in the United States. Individual participants were chosen that met the requirements to form a comparable German and U.S. sample. In the email that was sent to potential participants, we wrote: “In connection with Heidelberg University, we [names of authors] are currently conducting an international study to investigate the career path motivations of women and men after completion of their PhD work”.

The sample was recruited from five universities: For the German sample, we decided to include two high-ranking universities, one from former West Germany and one from former East Germany. We based this decision on the 2010 edition of the Shanghai ranking (http://www.arwu.org/), which compares and ranks the top five hundred universities in the world, based on criteria like number and citation rates of publications and scientific awards. For the U.S. sample, we selected one Ivy League university and two state universities; all of which ranked very highly in the mentioned ranking system.

We used the software “Soscisurvey” (www.soscisurvey.de), which offers all state of the art requirements for online based surveys. Each recipient was addressed personally via e-mail and received an individual serial number for the questionnaire. The aim of this procedure was twofold: first, it ensured that each participant could only fill in the questionnaire once. Second, it was possible to remind those recipients who had not responded to the survey. However, it was not possible to link the serial number to participant’s email addresses, therefore guaranteeing anonymity of responses. The e-mail addresses of the potential participants were collected individually from the universities’ home pages. Early career researchers from various academic fields were identified and addressed. A balanced number of men and women was equally attained. In the personalized email, it was stated that participation was a vital part of an international comparison study to investigate the work situation of early career researchers in Europe and in the United States. No incentives besides receiving the study’s findings after completion were offered to participants. Two reminders were sent to those participants who did not fill in the questionnaire after ten and fourteen days, respectively.

This procedure revealed the following total sample sizes and response rates (in parentheses): German university 1: *N* = 79 (36%), German university 2: *N* = 156 (35%), U.S. university 1: *N* = 83 (18.5%), U.S. university 2: *N* = 103 (22%), U.S. university 3: *N* = 42 (20%). The mean duration of filling in the questionnaire was *M* = 14.75 (*SD* = 4.4) minutes. From the initial total sample size of *N* = 463, we eliminated 13 participants who did not fill in any questionnaires (including demographic information). Fifty participants reported that they no longer worked in academia at the time of assessment and were also excluded; one more participant was excluded due to a nonsensical response (self-reported age older than 900 years). The final sample comprised 95 women and 107 men representing the two German universities, and 96 women and 101 men from three U.S. universities. The total sample size was *N* = 399.

We also assessed the academic fields of the study participants (by the question: “In which discipline did you acquire your PhD?”). We then classified these answers following the ISCED Fields of education and training 2013 [33], and formed four categorizations (arts and humanities; social sciences + business and law; natural sciences; and other academic fields). About 40% of participants in both countries acquired their PhD in natural sciences (36% in Germany, 42% in the US), about a quarter of participants in social sciences / business / law (Germany: 25%, US: 25%) and arts and humanities (Germany: 24%, US: 20%), respectively. There was no statistically meaningful difference between the two countries regarding academic fields, *χ*^*2*^(3) = 1.97, *p* = .579, Cramer’s *V* = .07. A detailed description of the sample is shown in S1 Table. The data can be retrieved from https://osf.io/qk3em/.

### Ethical statement

The Ethics Commission of the Faculty of Behavioural and Cultural Studies at Heidelberg University considers studies conducted by students as part of their degree program as exempt from ethical approval and expects the supervisors (in this case: Monika Sieverding, the first author) of the students to control that the ethical guidelines are fulfilled. Therefore, in their guidelines it is stated that students’ studies are only reviewed in in well-reasoned exemptions. The guidelines can be retrieved here (unfortunately only in German): http://www.verkult.uni-heidelberg.de/Formulare/Ethikkommission-Fakultaet-VerKult-HD_Informationen-zum-Verfahrensablauf.pdf). As the data collection for the study presented in this manuscript stems from the Master thesis of Thomas Stahl (the last author), and the study did not include any features that could be ethically relevant, we did not ask the Ethics Commission for ethical approval. The supervisor of the study (Monika Sieverding) confirms that the study was conducted in line with the ethical guidelines set out by the German Psychological Society (DGPs: https://www.dgps.de/fileadmin/documents/Empfehlungen/berufsethische_richtlinien_dgps.pdf. The study did not bear any obvious risk to the physical or psychological well-being of the participants involved.

All data were collected and treated anonymously. Participants were informed about the goal of the study, the procedures, and participants’ rights. Participation was voluntary. Subjects were informed that they could withdraw from the study at any point without repercussions. Subjects were assured that their data would be treated anonymously and that their answers could not be linked to them. Participants were also informed that they could always contact the PI at any time if they had questions about the study. Participants were informed that they are invited to participate in the study if they wish to by clicking on the link in the email. Informed consent was given by all participants. All data was treated anonymously. As the data were collected via Soscisurvey, there was no option to track individuals or see who they were. The IP addresses were not recorded.

### Measures

When clicking on the provided personalized link, informed consent was obtained. It was also possible to stop the survey at any given point and continue with the survey at a later point. A point of contact was also provided in case participants had questions.

*Work hours*. For assessing *actual* work hours, participants were asked: “Please think about your typical work week. How many total hours do you work currently in an average work week (regardless of where this work takes place)?”. *Ideal* work hours were assessed by asking: “What would be the ideal working hours for you per week?”. Both of these questions were adapted from the study of Lubinski and colleagues [34].

#### Covariates

*Calling*. Calling was assessed with five items from the “Pennsylvania Work Life Questionnaire” [31], ranging from “not at all” to “a lot”. Specifically, we asked the following five questions: “I enjoy talking about my work to others”, “My work is one of the most important things in my life”, “If I was financially secure, I would continue with my current line of work even if I was no longer paid”, “My work makes the world a better place”, “I would choose my current work life again if I had the opportunity”. The items were translated into German and then back-translated into English for verification by two bilingual persons. Cronbach’s alpha in the U.S. sample was .80 and in the German sample .72. Higher values indicate seeing the job as a calling.

*Mother-child ideology*. Mother-child ideology was assessed by the subscale “mother and child ideology” of the questionnaire “prejudices against workforce participation of women” [35]. The four items “A pre-school child is likely to suffer if his or her mother works”, “A mother is irreplaceable in the first year of a child's life”, “Women, who are especially strong in terms of their engagement in their professions, cannot be good mothers at the same time”, “Mothers of small children, who pursue their professions full-time, do so at the expense of their children's development” were measured on a 5-point Likert-scale ranging from “strongly disagree” to “strongly agree”. The items were translated into English and then back-translated into German for verification by two bilingual persons. Cronbach’s alpha in the U.S. sample was .76 and in the German sample .81. Higher values indicate more traditional mother-child attitudes.

*Gender* was coded with 0 (male) and 1 (female). *Years since PhD* was calculated from answers given to the question “When did you finish your PhD (thesis defense or viva voce, respectively)?”. *Duration of PhD* was assessed with the question “In total, how long did it take you to finish your PhD (in years)?” and here it was possible to answer in decimals. *Parenthood* was assessed with the question “Do you have children?” (yes; coded as 1 / no; coded as 0). Participants’ country was coded as 0 (Germany) or 1 (USA), respectively.

#### Additional variables

We also examined whether the effects remained unchanged even after controlling for further variables. We note that in contrast to the covariates specified in the previous section, these variables were not chosen as independent variables in an a-priori fashion. Results involving these variables should therefore be interpreted as exploratory. The main reason for including these variables was to examine if the effects were robust when including additional independent variables. *Perceived career attractiveness* was measured using one item (“How attractive do you consider an academic career for you personally?”) with responses ranging from 1 “very unattractive” to 5 “very attractive”. *Distribution of work time* was assessed as the percentage of work time devoted to research, teaching, and administration, respectively (with the constraint that percentages needed to amount to 100%). Further, we assessed whether the participants’ current position was *permanent* (coded as 1) or not (coded as 0). Results involving these variables will be presented at the end of the Results section.

### Data analysis

Descriptive statistics for the sample data are presented in the Supplementary S1 and S2 Tables. Three hundred and forty-seven participants (87% of the sample) had complete data on all study variables. Missing values were replaced using multiple imputation implemented via the mice package [36] in R. Thirty imputations were computed and the pooled results across the imputed data sets are reported. Note that this resulted in non-integer values for degrees of freedom. The code necessary to reproduce the reported results can be retrieved from https://osf.io/qk3em/.

## Results

Descriptive statistics of the study variables are displayed in S1 Table. Product-moment correlations of the relevant study variables are depicted in S2 Table (note that unlike the results presented in the next sections, these descriptive statistics are based on the non-imputed data).

### Actual work hours

In line with prior research, we expected that early career researchers working in Germany would report shorter work hours than early career researchers in the US, which was supported, *t*(373.9) = 5.57, *p* < .001, *d* = 0.56 (*M*_Germany_ = 45.2, *SD*_Germany_ = 10.3; *M*_US_ = 51.5, *SD*_US_ = 11.9). Comparing the work hours of men and women, results revealed that female researchers reported shorter work hours than male researchers, *t*(388.2) = 2.76, *p* = .006, *d* = 0.28. However, separating the analyses by Country showed that this effect was statistically significant only in the German sample, *t*(190.9) = 3.81, *p* < .001, *d* = 0.54 (*M*_women_ = 42.3, *SD*_women_ = 11.5; *M*_men_ = 47.8, *SD*_men_ = 8.3), but not in the U.S. sample, *t*(187.2) = 0.65, *p* = .516, *d* = 0.09 (*M*_women_ = 50.9, *SD*_women_ = 11.4; *M*_men_ = 52.1, *SD*_men_ = 12.3).

We found an overall statistically significant main effect of Parenthood on reported actual work hours, such that early career researchers with children reported shorter work hours than those without children, *t*(371.6) = 5.15, *p* < .001, *d* = 0.52. We expected that the effect of Parenthood on work hours would be moderated by Gender and Country, such that the effect of Parenthood on work hours would be stronger for women than for men and attenuated for women from the US compared with women from Germany.

To examine the interactive effect of Gender, Parenthood, and Country, we conducted a multiple regression analysis predicting reported work hours. In the first model (see Model 1, Table 1), only the four covariates (Calling, Mother-child ideology, Duration of PhD and Years since PhD) were included as independent variables. These independent variables were standardized (z-transformed) for the analyses. This model explained 7% of the variance in self-reported work hours. Calling, *b* = 2.94, *p* < .001, and years since PhD, *b* = -1.43, *p* = .015, predicted actual work hours with higher calling and less years since completion of the PhD being associated with more work hours.

**Table 1:** Regression analyses explaining actual work hours (left columns) and ideal work hours (right columns).

	Actual Work Hours		Ideal Work Hours	
	Model 1	Model 2	Model 3	Model 4
Intercept	48.32*** (0.57)	48.59*** (1.37)	41.08*** (0.45)	40.71*** (1.09)
Calling	2.94*** (0.58)	2.48*** (0.55)	2.86*** (0.45)	2.60*** (0.43)
Mother-Child Ideology	-0.54 (0.58)	-0.85 (0.54)	-0.37 (0.45)	-0.66 (0.42)
Duration PhD	-0.71 (0.57)	-1.62** (0.55)	-0.19 (0.45)	-0.84 (0.44)
Years since PhD	-1.43* (0.59)	-0.17 (0.58)	-1.51** (0.56)	-0.61 (0.46)
Gender^a^	-	-1.22 (2.07)	-	0.18 (1.64)
Parenthood^b^	-	-2.16 (2.03)	-	-1.56 (1.60)
Country^c^	-	6.17** (1.90)	-	6.17*** (1.51)
Gender x Parenthood	-	-7.19* (2.94)	-	-5.42* (2.32)
Gender x Country	-	-1.24 (2.86)	-	-4.93* (2.29)
Parenthood x Country	-	-4.39 (2.95)	-	-1.26 (2.33)
Gender x Parenthood x Country	-	10.67* (4.20)	-	6.30 (3.36)
***R***^***2***^ **(adjusted)**	**.07**	**.22**	**.11**	**.24**

Note. *N* = 399. Table depicts unstandardized regression coefficients (standard errors in parentheses). Reported results are pooled estimates across 30 imputed data sets. Adjusted *R*^*2*^ is the mean adjusted *R*^*2*^ across the 30 imputed data sets. Continuous independent variables were z-standardized prior to the analysis.

*p < .05.

**p < .01.

***p < .001.

^a^0 = male, 1 = female.

^b^0 = no children, 1 = children.

^c^0 = Germany, 1 = USA.

In the next model (Model 2; Table 1), we further added the three focal independent variables (Gender, Parenthood, and Country) as well as the two-way interactions and the three-way interaction of these three variables. The three variables were dummy coded and the reference categories were men, participants without children and participants from Germany. Including these independent variables into the model increased explained variance (*R*^*2*^) to 22%. Results revealed a statistically significant conditional main effect of Country, *b* = 6.17, *p* = .001, a statistically significant conditional Gender x Parenthood interaction, *b* = -7.19, *p* = .015, and a statistically significant Gender x Parenthood x Country interaction, *b* = 10.67, *p* = .011. In the presence of higher order interactions, the conditional main effect of Country indicates that men without children in the U.S. sample worked approximately 6.2 hours more than men without children in the German sample. The Gender x Parenthood interaction indicates that in the German sample differences in self-reported work hours between men and women were approximately 7.2 hours larger among parents compared to participants who did not have children.

**Fig 1:**
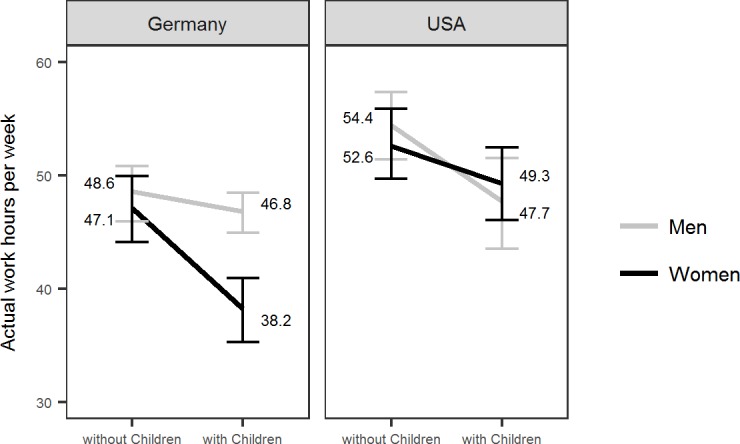
Actual work hours of German and U.S. early career researchers as a function of gender and parenthood. Figure depicts the empirical (not imputed) means. Errorbars indicate 95% bootstrap confidence intervals.

Finally, the Gender x Parenthood x Country interaction was positive, showing that the (negative) Gender x Parenthood interaction in the German sample was absolutely smaller (in fact even slightly positive) in the U.S. sample. To further illuminate this interaction, we split the data by Country and predicted work hours by the four covariates (standardized by Country) as well as the independent variables Gender and Parenthood and the Gender x Parenthood interaction (see Table 2 for results). The model explained 9% of the variance in self-reported work hours in the U.S. sample and 23% of the variance in the German sample. In the U.S. sample, there was a statistically significant conditional main effect of Parenthood, *b* = -6.35, *p* = .012, indicating that men with children reported about 6.4 less work hours than men without children. There was no statistically significant Gender x Parenthood interaction in the U.S. sample, *b* = 3.37, *p* = .317, providing no evidence for a larger effect of parenthood for women than for men. In the German sample, there was a statistically significant Gender x Parenthood interaction, *b* = -7.26, *p* = .006, indicating a larger difference between parents and participants without children for women than for men. The mean levels of reported actual work hours are depicted in Fig 1 (the boxplots for actual work hours separately for these eight groups can be found in the supplemental material, S1 Fig). As can be seen from this illustration, German female researchers with children worked about 8 fewer hours in a week than their male counterparts, whereas in the US, there was no discernible difference in work hours between men and women with children. These results clearly support the “*mothers work less*” hypothesis for German but not for U.S. early career researchers.

**Table 2:** Regression analyses by Country explaining actual work hours (left columns) and ideal work hours (right columns).

	Actual Work Hours		Ideal Work Hours	
	USA	Germany	USA	Germany
Intercept	54.63*** (1.23)	48.72*** (1.23)	46.72*** (1.28)	40.64*** (0.90)
Calling	2.63** (0.85)	2.37*** (0.69)	2.75*** (0.70)	2.47*** (0.50)
Mother-Child Ideology	-0.06 (0.93)	-1.43* (0.62)	-0.25 (0.77)	-0.91* (0.45)
Duration PhD	-1.84* (0.89)	-1.36* (0.67)	-0.27 (0.74)	-1.33** (0.48)
Years since PhD	-0.24 (1.00)	0.11 (0.67)	-0.62 (0.84)	-0.60 (0.48)
Gender^a^	-2.22 (2.21)	-1.30 (1.84)	-4.77* (1.86)	0.04 (1.34)
Parenthood^b^	-6.35* (2.49)	-2.18 (1.80)	-2.91 (2.08)	-1.59 (1.31)
Gender x Parenthood	3.37 (3.35)	-7.26** (2.62)	0.94 (2.82)	-5.45** (1.89)
***R***^***2***^ **(adjusted)**	**.09**	**.23**	**.12**	**.27**

Note. *N* = 197 (USA); *N* = 202 (Germany). Table depicts unstandardized regression coefficients (standard errors in parentheses). Reported results are pooled estimates across 30 imputed data sets. Adjusted *R*^*2*^ is the mean adjusted *R*^*2*^ across the 30 imputed data sets. Continuous independent variables were z-standardized prior to the analysis.

*p < .05.

**p < .01.

***p < .001.

^a^0 = male, 1 = female.

^b^0 = no children, 1 = children.

### Ideal work hours

We repeated the analyses reported above, changing the dependent variable to ideal work hours. As in previous analyses, statistically significant differences emerged between researchers from the US and Germany (*M*_Germany_ = 38.5, *SD*_Germany_ = 7.7; *M*_US_ = 43.7, *SD*_US_ = 10.1; *t*(386.4) = 5.75, *p* < .001, *d* = 0.58), between men and women (*M*_women_ = 39.2, *SD*_women_ = 8.6; *M*_men_ = 42.8, *SD*_men_ = 9.6; *t*(382.3) = 3.99, *p* < .001, *d* = 0.40), and between researchers with versus without children (*M*_with children_ = 38.8, *SD*_with children_ = 9.3; *M*_without children_ = 43.0, *SD*_without children_ = 8.9; *t*(374.1) = 4.56, *p* < .001, *d* = 0.46), respectively. In contrast to the results on actual work hours, the gender difference in ideal work hours was statistically significant in both the German (*M*_women_ = 36.7, *SD*_women_ = 7.6; *M*_men_ = 40.0, *SD*_men_ = 7.4; *t*(194.6) = 3.09, *p* = .002, *d* = 0.44) and the U.S. sample (*M*_women_ = 41.5, *SD*_women_ = 8.9; *M*_men_ = 45.8, *SD*_men_ = 10.7; *t*(184.5) = 2.99, *p* = .003, *d* = 0.43).

**Fig 2:**
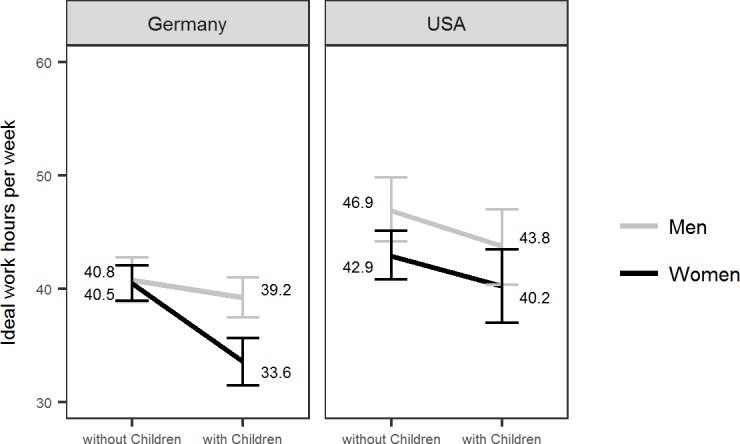
Ideal work hours of German and U.S. early career researchers as a function of gender and parenthood. Figure depicts the empirical (not imputed) means. Errorbars indicate 95% bootstrap confidence intervals.

Results from the multiple regression (Table 1, right column) showed a statistically significant main effect of Country, *b* = 6.17, *p* < .001, a statistically significant Gender x Country interaction, *b* = -4.93, *p* = .032, and a statistically significant Gender x Parenthood interaction, *b* = -5.42, *p* = .020. The Gender x Parenthood x Country interaction failed to reach statistical significance, *b* = 6.30, *p* = .062. Given our a-priori hypothesis regarding differences in the Gender x Parenthood interaction between the two countries, we nevertheless split the sample by country and investigated the Gender x Parenthood interaction separately for the two countries. Results (see Table 2) revealed a similar pattern for ideal work hours as for actual work hours: Only in the German sample, *b* = -5.45, *p* = .004, but not in the U.S. sample, *b* = 0.94, *p* = .738, was there a statistically significant Gender x Parenthood interaction, indicating support for a “*mothers want to work less*” hypothesis in Germany. In the U.S. sample, only the main effect of Gender was statistically significant, *b* = -4.77, *p* = .011, indicating that women overall (regardless of parental status) reported on average lower ideal work hours than men in the US (as illustrated Fig 2).

### Additional analyses

In a last step, we investigated whether findings were robust when controlling for further third variables in addition to the a-priori specified covariates. Specifically, we investigated perceived career attractiveness, distribution of work time, and whether participants’ current position is permanent or not. First, we tested whether there were statistically meaningful effects of Gender, Parenthood, or Country on these variables. For Career attractiveness, none of the main effects or interaction effects surpassed the threshold for statistical significance, *p* > .075 for all. For Distribution of work time, there were statistically significant main effects of Country for Percent of work time devoted to research, *b* = 15.4, *p* = .002, and Percent of work time devoted to teaching, *b* = -10.2, *p* = .014, but not Percent of work time devoted to administrative duties, *b* = -5.2, *p* = .073. Compared to their U.S. colleagues, German early career researchers devoted a smaller proportion of their time to research activities (*M*_Germany_ = 51.0%, *SD*_Germany_ = 26.2%; *M*_US_ = 58.2%, *SD*_US_ = 27.7%) and more time to teaching (*M*_Germany_ = 32.4%, *SD*_Germany_ = 22.4%; *M*_US_ = 27.4%, *SD*_US_ = 22.9%). No other main effects or interactions were statistically significant in predicting Distribution of work time, *p* > .067 for all. When predicting the odds of reporting a Permanent position from Gender, Parenthood, and Country in a logistic regression model, only the main effect for Country was statistically significant, *b* = 2.57, *p* < .001 (*p* > .104 for all other effects): Early career researchers in the US more often reported having a permanent position (55.8%) compared to researchers in Germany (15.7%), odds ratio = 6.77.

Including the independent variables Permanent position, Career attractiveness, Proportion of time devoted to research, and Proportion of time devoted to teaching as additional covariates into the regression models (Model 2 and Model 4) did not change the pattern of results (note that % of time devoted to administration was excluded since including all three work time distribution variables would result in singularity among independent variables). Specifically, for Actual work hours, the main effect for Country, *b* = 4.69, *p* = .022, the Gender x Parenthood interaction, *b* = -6.73, *p* = .022, and the Gender x Parenthood x Country interaction, *b* = 9.63, *p* = .022, remained statistically significant. For Ideal work hours, the main effect for Country, *b* = 4.73, *p* < .001, and the Gender x Parenthood interaction, *b* = -5.33, *p* = .022, remained statistically significant, while the Gender x Parenthood x Country interaction again failed to reach statistical significance, *b* = 5.90, *p* = .080. Detailed results can be found in S3 Table (Model 5 and Model 7). Excluding all covariates and keeping only the main effects and interactions of Gender, Parenthood, and Country did not change the pattern of results either (see Model 6 and Model 8 in S3 Table). Furthermore, the predicted group means remained unaltered by the inclusion (Model 5 and Model 7) or exclusion (Model 6 and Model 8) of covariates (see S4 Table). Hence, the results reported seemed robust to alterations in the included covariates.

#### Power

The sample size for the present study was not determined by a formal a-priori power analysis. A post-hoc sensitivity analysis was run in G*Power [37] to determine the lower boundaries of effect sizes that could be detected as statistically significant in our sample (assuming an alpha error of .05 and power of .95). For mean comparisons between two groups using the full sample (e.g., Germany vs. US), these analyses revealed effect sizes of *d* = .37 of higher; for the multiple regression analysis, these analyses yielded an effect size of *f*^*2*^ = .04 or higher. Hence, the present study was adequately powered (power = 95%) to detect effect sizes which are typically considered small to medium [38] as statistically significant.

## Discussion

While there is profound evidence for gender discrimination in the past, nowadays the gender gap in academia may rather–at least partly–stem from gender differences in lifestyle preferences [6, 8, 5, 4, 7]. The aim of our study was to investigate work hours of early career researchers, using a German and U.S. sample, which may add to explaining the remaining gender gap in academia.

Consistent with other research, this study found that U.S. early career researchers work more hours than German researchers. Results showed that female German early career researchers (on average) worked less than male German researchers. Additionally, results supported the “*mothers work less*” hypothesis in Germany, such that female researchers (on average) worked substantially less when they had children. This supports the notion that “babies do matter” [39] for female researchers in Germany. As work hours are an important indicator of vocational engagement and a predictor of vocational success [11], the differences in work hours may have important consequences for the future of women in academia. The present study did not find evidence for a similar effect for the U.S. sample [25]. Specifically, in the US, male and female researchers (on average) worked equal amount of hours, and this effect held when researchers had children.

Results remained similar when analyzing ideal working hours, showing: On average, U.S. researchers wanted to work more than their German counterparts, men wanted to work more than women, researchers with children wanted to work less than those without children. These results support the research by Ferriman et al. [8] and Lubinski et al. [13] who suggested that male and female academics have different priorities with men putting more focus on their work life. In Germany, the additional effect of “*mothers want to work less*” emerged such that female researchers with children in Germany (on average) wanted to work less than male researchers with children. The analyses of ideal work hours provide additional support for our hypothesis that there are gender differences in lifestyle preferences. In Germany, mothers not only worked less on average but they also *wanted* to work less compared to male counterparts and comparable female researchers in the US. Therefore, lifestyle preferences that are associated with parenthood may explain the reduced work hours of women in academia in Germany.

In this study, we found striking differences in hours worked between comparable German and U.S. early career researchers. It may well be that U.S. employees have higher pressure to put in long hours [17]. Anecdotal evidence exists that suggests female academics in the US struggle considerably to balance the demands of a long hour culture with demands of having children. Although there was no evidence for a gender difference in actual working hours in the U.S. sample, we found a small gender difference in ideal work hours, such that female U.S. researchers wanted to work fewer hours than their male counterparts. This pattern of results may indicate that women in the US adapt to the norm of a long hours culture rather than fulfil their ideal working arrangement.

### Potential implications for research productivity

When looking at the hours worked across the two samples, the difference in result patterns between Germany and the US is striking. Relating back to the argument made by Lubinski [22] that accumulated gender differences in hours over time may have negative consequences for women, we made a rough calculation on the discrepancy over a period of five years. Female early career researchers with children in Germany reported working 38.2 hours per week whereas their male counterparts reported working 46.8 hours per week. Extrapolating from this information results in a difference of roughly 1,978 hours worked more by male researchers in Germany than female researchers in Germany over a period of five years (We took the difference in actual hours worked between female and male researchers with children (8.6) multiplied by 46 (52 weeks in a year minus 6 weeks of annual leave) multiplied by 5 years). Arguably, the first five years after the PhD are crucial for the career prospects in academia. The question is how many (more) papers and grant proposals can one write in 1,978 hours? A recent study investigated the representation of female authorships in high quality research from 2008 to 2016 and found that women were underrepresented at prestigious authorships compared to men [40]. It is not surprising that researchers, publishers, and faculty selection committees ask themselves: “Where are the Women?” [41–43].

Are (female) researchers with children less productive than researchers without children? Results regarding the relationship between children and research productivity of women and men are inconsistent [see overview in 44]. Reviewing the evidence until 1980, studies showed either no effect or a negative effect of children on the productivity of women [12]. Fox [45, 46], however, found a positive effect of having children on publication record. The author speculated whether there might be a selection effect operating, such that women in academia who do have children may have greater health and energy than women without children, or whether women deliberately delay having children until they have established research productivity. Cole and Zuckerman [47] interviewed 120 American scientists and found that men generally published more, yet that women published as much with or without children. Hunter and Leahey analyzed the effects of children on entire careers of academics and found that children had a negative effect on the rate of productivity growth, and this negative trend was more pronounced among women [44]. The authors speculate that the division of labor at home may be a determining factor for this gender difference. Similarly, a study among Norwegian academics in 1992 revealed that children affected the research productivity of women but not men [48], and the authors suggested that career interruptions due to childbirth and caring responsibilities decreased the productivity of women. A recent German survey explored the association between gender and research productivity during the doctoral phase. The analyses revealed that women published less than men. However, in contrast to the author’s initial hypotheses, there were no gender-specific effects of parenthood on scholarly productivity [49]. In sum, it seems safe to postulate that it is not parenthood itself that exerts a negative influence on women’s productivity. Factors that can explain lower research productivity of female researchers may include reduced vocational engagement like reduced working hours [26], interrupted careers, or timing aspects, childcare provisions, and partner’s involvement in childcare.

### Psychological and cultural aspects

Our results might help explain the difference in the percentage of female professorships in the US and Germany. Whereas nowadays every third full professor in the US is female, this number is still much smaller in Germany where only every fifth professor is female. Traditional gender roles that expect women to interrupt or reduce their vocational careers when they have a child are more common in Germany [30]. Mothers of small children in Germany are still confronted with the risk of being branded as bad mothers or “raven mothers” when they continue with their careers [50]. One could argue that the cultural pressure–especially the discourse on work and parenthood–in Germany is so strong that it is not an individual’s decision to work less as a mother. Such discourses influence the individuals on two levels: the extent to which they internalize these values and the extent to which they do not internalize them but are still bound by them. In our study, we tried to control for the individual level by measuring work as a calling and mother-child ideology. As after controlling for these individual values we still found an effect of country and an interaction between gender, parenthood and country, there remains an effect independent of the internalization of these values on the individual level. It is very probable that the mother-child ideology scale is not capable to assess the full amount of internalization of the cultural pressure on women with children in Germany. Another possibility is that young and qualified mothers in Germany feel forced to reduce their work hours because they still carry the main burden of family and household work [51]. However, the fact that we found a very similar result pattern in the ‘ideal hours’ speaks against this latter interpretation. Young female researchers in Germany work less *and* want to work less than their male counterparts and less than their colleagues in the US. Additionally, the parental leave policies along with other German tax legislations that reward the reduced work hours and interruption of careers of married women (“Ehegattensplitting” = “income tax splitting”) might have unintended boomerang effects as they can knock women out of the system and reduce their chances of having a successful career.

Related to this, we want to speculate about a potential ‘psychological motherhood penalty’, such that women may (fear to) be subjected to discrimination of being a mother. Corell et al. [52] showed that mothers received lower ratings on competence, commitment and were less likely to be recommended to hire. In one study on junior faculty members, it was found that having children was associated with being perceived by senior faculty as less committed and involved, less flexible, and as having less desire for advancement–but this was only true for female faculty, not for male faculty [53]. It may be that German and U.S. mothers are subject to different kinds of fears. Female German researchers with children may fear most that they will be seen as ‘bad mothers’. As a result, they reduce their work hours, as seen in this study. Female U.S. researchers with children, however, may fear more to be seen as ‘bad researchers’, and may, therefore, not reduce their work hours but potentially may spend less time with their children. For instance, a study from the US found that female faculty members reported having fewer children than desired compared to male faculty members [54].

### Limitations

Like any study, ours is not without limitations. One limitation relates to our samples. First, both samples are based on convenience sampling. However, it seems likely that our sampling strategy resulted in conservative estimates, such that early career researchers who work more hours likely did not find the time to participate in our study. Second, we sampled from universities with high levels of reputation, which may limit the generalization of our results to early career researchers in those kinds of universities. While we achieved comparability in the recruitment method and demographic variables (e.g. age, years since PhD, parenthood) between the German and the U.S. sample, the U.S. researchers had a higher percentage of permanent jobs.

Other limitations relate to our measure of work hours. We asked respondents to judge their own work hours. Thus, it is unclear whether similar effects would be found for objectively measured work hours. Moreover, work hours are only a proxy of success. Therefore, it remains an open question whether work hours actually mediate the effects of parenthood on future success like tenure, becoming a professor, or number and quality of publications.

One could make the argument that women (and men) caring for children may just work smarter rather than longer, using their limited time to focus on what they are really interested in and avoiding non-productive tasks like administrative tasks. Indeed, Fox [46] found that women with pre-school children spent more time advising graduate students than undergraduate students. In our study, we only asked for time devoted to research, teaching and administrative tasks, and found no differences as a function of gender or parenthood. Nevertheless, there might be qualitative differences in the way men and women with versus without children use their work hours that have not been addressed in this study. Therefore, future studies may want to include a more detailed assessment of work-related aspects beyond the mere quantitative amount of work hours.

Moreover, we want to mention that this study is based on a cross-sectional design. Therefore, it is unclear if parenthood causally impacts work hours or whether there are potential other third variables that impact both work hours and the likelihood of parenthood outside of the covariates we studied.

One other limitation refers to the fact that we did not measure the age of participants’ children. We would expect that researchers with very young children may have different work hours than researchers with older children. Mason [55] argued that the timing of children is important for the career prospects of early career researchers. Similarly, Hunter & Leahey [44] argued that children of different ages demand different amount of time. They reported that the productivity of academics increased in the first year of a child and again when the child entered school. However, in general, they report a negative effect of children on productivity growth.

Lastly, the regression analyses showed that variance explanation was bigger in Germany (21–27%) than in the US (7–11%) suggesting that there are additional important predictors (in particular in the U.S. sample) that we have not included in the current study.

## Conclusions

Our study investigated early career researchers in the US and in Germany. The hypothesis that female researchers with children work less and want to work less than female researchers without children and male researchers with children was supported for the German but not for the US sample. For participants from the US, we found a small gender difference in ideal work hours such that female researchers would ideally want to work less than male researchers but no gender difference in actual work hours. Our results indicate that gendered work hours are no longer capable of explaining the gender gap in higher positions in academia in the US. It will be interesting to investigate the “*mothers work less*” hypothesis among early career researchers in other countries with different gender role arrangements and different (parental leave and tax) legislations.

## Supporting information

S1 Table Summary of descriptive statistics. Note. Table depicts means (*M*) and standard deviations (*SD*) separately for men and women, early career researchers with and without children, and early career researchers from Germany and the United States, respectively.
(DOCX)

S2 Table Intercorrelations of study variables. Note. N = 352–399. Table depicts product-moment correlations of the study variables. *p < .05; **p < .001.
(DOCX)

S3 Table Regression analyses explaining actual work hours (left columns) and ideal work hours (right columns). Note. N = 399. Table depicts unstandardized regression coefficients (standard errors in parentheses). Reported results are pooled estimates across 30 imputed data sets. Adjusted R^2^ is the mean adjusted R^2^ across the 30 imputed data sets. Continuous independent variables were z-standardized prior to the analysis. *p < .05; **p < .01; ***p < .001.
(DOCX)

S4 Table Predicted means in actual work hours (left columns) and ideal work hours (right columns), depending on the included covariates. Note. Table depicts the predicted means in actual and ideal work hours from the models including the a-priori covariates (Model 2 and Model 4; Table 1), the models including no covariates (Model 6 and Model 8; S3 Table), and the models including all reported independent variables (Model 5 and Model 7; S3 Table).
(DOCX)

S1 Fig Boxplots of actual work hours of German and U.S. early career researchers as a function of gender and parenthood. (TIFF)
