# Supplementary material for: Can lifestyle preferences help explain the persistent gender gap in academia? The “mothers work less” hypothesis supported for German but not for U.S. early career researchers
Source: PLoS One. 2018 Aug 28;13(8):e0202728. doi: 10.1371/journal.pone.0202728 (PMC6112653; doi:10.1371/journal.pone.0202728)
Supplement: S1 Table — Note. Table depicts means (M) and standard deviations (SD) separately for men and women, early career researchers with and without children, and early career researchers from Germany and the United States, respectively. (DOCX) [file pone.0202728.s001.docx]

**S1 Table. Summary of Descriptive Statistics.**

|  | **Germany** | | | | | | | | | **USA** | | | | | | | | |
| --- | --- | --- | --- | --- | --- | --- | --- | --- | --- | --- | --- | --- | --- | --- | --- | --- | --- | --- |
|  | Men | | | | | Women | | | | Men | | | | Women | | | | |
|  | No children | | Children | | | No children | | Children | | No children | | Children | | No children | | Children | | |
|  | *M* | *SD* | | *M* | *SD* | *M* | *SD* | *M* | *SD* | *M* | *SD* | *M* | *SD* | *M* | *SD* | *M* | *SD* |  |
| Age | 36.0 | 4.8 | | 39.5 | 7.3 | 36.1 | 7.4 | 41.9 | 8.7 | 34.3 | 5.3 | 39.5 | 8.3 | 36.1 | 5.5 | 42.0 | 6.7 |  |
| Years since PhD | 4.1 | 3.1 | | 7.4 | 6.9 | 4.3 | 4.4 | 9.6 | 8.6 | 3.7 | 2.6 | 8.0 | 7.7 | 5.3 | 4.8 | 8.0 | 4.6 |  |
| Duration PhD | 4.7 | 2.1 | | 4.6 | 1.1 | 4.5 | 1.3 | 4.4 | 1.3 | 5.2 | 1.2 | 5.7 | 1.7 | 5.5 | 1.5 | 5.7 | 1.5 |  |
| Calling | 2.9 | 0.6 | | 2.9 | 0.7 | 2.7 | 0.6 | 2.8 | 0.7 | 2.9 | 0.8 | 3.0 | 0.7 | 3.1 | 0.6 | 3.1 | 0.6 |  |
| Mother-Child Ideology | 2.2 | 0.9 | | 2.2 | 1.1 | 2.1 | 1.2 | 1.9 | 0.9 | 2.2 | 0.8 | 2.1 | 1.0 | 2.0 | 0.8 | 2.0 | 0.8 |  |
| Actual work hours | 48.6 | 9.5 | | 46.8 | 6.2 | 47.1 | 10.3 | 38.2 | 10.8 | 54.4 | 11.7 | 47.7 | 12.3 | 52.6 | 11.4 | 49.3 | 11.5 |  |
| Ideal work hours | 40.8 | 8.0 | | 39.2 | 6.8 | 40.5 | 5.4 | 33.6 | 7.7 | 46.9 | 11.0 | 43.8 | 9.8 | 42.9 | 7.5 | 40.2 | 10.3 |  |

Note*.* Table depicts means (*M*) and standard deviations (*SD*) separately for men and women, early career researchers with and without children, and early career researchers from Germany and the United States, respectively.
