# Supplementary material for: Can lifestyle preferences help explain the persistent gender gap in academia? The “mothers work less” hypothesis supported for German but not for U.S. early career researchers
Source: PLoS One. 2018 Aug 28;13(8):e0202728. doi: 10.1371/journal.pone.0202728 (PMC6112653; doi:10.1371/journal.pone.0202728)
Supplement: S2 Table — Note. N = 352–399. Table depicts product-moment correlations of the study variables. *p < .05; **p < .001. (DOCX) [file pone.0202728.s002.docx]

**S2 Table. Intercorrelations of Study Variables.**

|  | **(2)** | **(3)** | **(4)** | **(5)** | **(6)** |
| --- | --- | --- | --- | --- | --- |
| (1) Years since PhD | -.06 | .07 | -.06 | -.10 | -.14* |
| (2) Duration PhD |  | .05 | -.02 | -.04 | .01 |
| (3) Calling |  |  | -.13* | .26** | .30** |
| (4) Mother-Child Ideology |  |  |  | -.07 | -.08 |
| (5) Actual work hours |  |  |  |  | .59** |
| (6) Ideal work hours |  |  |  |  |  |

Note*.* *N* = 352-399. Table depicts product-moment correlations of the study variables. **p* < .05; ***p* < .001.
