# Supplementary material for: Can lifestyle preferences help explain the persistent gender gap in academia? The “mothers work less” hypothesis supported for German but not for U.S. early career researchers
Source: PLoS One. 2018 Aug 28;13(8):e0202728. doi: 10.1371/journal.pone.0202728 (PMC6112653; doi:10.1371/journal.pone.0202728)
Supplement: S3 Table — Note. N = 399. Table depicts unstandardized regression coefficients (standard errors in parentheses). Reported results are pooled estimates across 30 imputed data sets. Adjusted R2 is the mean adjusted R2 across the 30 imputed data sets. Continuous independent variables were z-standardized prior to the analysis. *p < .05; **p < .01; ***p < .001. (DOCX) [file pone.0202728.s003.docx]

**S3 Table. Regression Analyses Explaining Actual Work Hours (left columns) and Ideal Work Hours (right columns).**

|  | **Actual Work Hours** | | **Ideal Work Hours** | |
| --- | --- | --- | --- | --- |
|  | Model 5 | Model 6 | Model 7 | Model 8 |
| Intercept | 48.39*** (1.39) | 48.63*** (1.40) | 40.75*** (1.10) | 40.76*** (1.13) |
| Calling | 2.01** (0.65) | **-** | 2.05*** (0.51) | **-** |
| Mother-Child Ideology | -0.88 (0.54) | **-** | -0.74 (0.43) | **-** |
| Duration PhD | -1.58** (0.55) | **-** | -0.82 (0.44) | **-** |
| Years since PhD | -0.55 (0.63) | **-** | -0.75 (0.50) | **-** |
| Permanent position^a^ | 2.39 (1.41) | - | 1.51 (1.10) | - |
| % time (research) | -0.93 (0.99) | - | 0.66 (0.76) | - |
| % time (teaching) | -1.64 (0.98) | - | -0.34 (0.75) | - |
| Career attractiveness | 0.52 (0.66) | - | 0.82 (0.53) | - |
| Gender^b^ | -1.39 (2.06) | -1.51 (2.14) | 0.19 (1.64) | -0.30 (1.72) |
| Parenthood^c^ | -2.29 (2.02) | -1.91 (2.09) | -1.66 (1.60) | -1.60 (1.67) |
| Country^d^ | 4.69* (2.04) | 5.94** (1.96) | 4.73** (1.63) | 6.25*** (1.58) |
| Gender x Parenthood | -6.73* (2.94) | -6.82* (3.05) | -5.33* (2.31) | -5.22* (2.44) |
| Gender x Country | -0.58 (2.86) | -0.48 (2.94) | -4.47 (2.29) | -3.99 (2.39) |
| Parenthood x Country | -4.03 (2.95) | -4.95 (3.04) | -0.84 (2.31) | -1.66 (2.45) |
| Gender x Parenthood x Country | 9.63* (4.20) | 10.18* (4.32) | 5.90 (3.36) | 5.97 (3.52) |
| ***R*² (adjusted)** | **.22** | **.15** | **.25** | **.15** |

Note*.* *N* = 399. Table depicts unstandardized regression coefficients (standard errors in parentheses). Reported results are pooled estimates across 30 imputed data sets. Adjusted *R*² is the mean adjusted *R*² across the 30 imputed data sets. Continuous independent variables were z-standardized prior to the analysis. **p* < .05; ***p* < .01; ****p* < .001. ^a^0 = no permanent position, 1 = permanent position.
^b^0 = male, 1 = female.
^c^0 = no children, 1 = children.
^d^0 = Germany, 1 = USA.
