# Supplementary material for: Can lifestyle preferences help explain the persistent gender gap in academia? The “mothers work less” hypothesis supported for German but not for U.S. early career researchers
Source: PLoS One. 2018 Aug 28;13(8):e0202728. doi: 10.1371/journal.pone.0202728 (PMC6112653; doi:10.1371/journal.pone.0202728)
Supplement: S4 Table — Note. Table depicts the predicted means in actual and ideal work hours from the models including the a-priori covariates (Model 2 and Model 4; Table 1), the models including no covariates (Model 6 and Model 8; S3 Table), and the models including all reported independent variables (Model 5 and Model 7; S3 Table). (DOCX) [file pone.0202728.s004.docx]

**S4 Table. Predicted Means in Actual Work Hours (left columns) and Ideal Work Hours (right columns), Depending on the Included Covariates.**

|  | **Germany** | | | | | **USA** | | | |
| --- | --- | --- | --- | --- | --- | --- | --- | --- | --- |
|  | Men | | | Women | | Men | | Women | |
|  | No children | Children | | No children | Children | No children | Children | No children | Children |
|  | Actual work hours | | | | | | | | |
| No covariates  (Model 6) | 48.63 | | 46.71 | 47.12 | 38.34 | 54.57 | 47.70 | 52.58 | 49.07 |
| A-priori covariates  (Model 2) | 48.59 | | 46.43 | 47.37 | 38.02 | 54.76 | 48.20 | 52.30 | 49.23 |
| All covariates  (Model 5) | 49.24 | | 46.95 | 47.84 | 38.82 | 53.93 | 47.61 | 51.95 | 48.53 |
|  | Ideal work hours | | | | | | | | |
| No covariates  (Model 8) | 40.76 | | 39.17 | 40.47 | 33.64 | 47.01 | 43.76 | 42.72 | 40.21 |
| A-priori covariates  (Model 4) | 40.71 | | 39.15 | 40.89 | 33.91 | 46.88 | 44.06 | 42.13 | 40.19 |
| All covariates  (Model 7) | 41.29 | | 39.63 | 41.48 | 34.49 | 46.02 | 43.52 | 45.62 | 43.70 |

Note*.* Table depicts the predicted means in actual and ideal work hours from the models including the a-priori covariates (Model 2 and Model 4; Table 1), the models including no covariates (Model 6 and Model 8; Table S3), and the models including all reported independent variables (Model 5 and Model 7; Table S3).
