# Supplementary figures and images for: Can lifestyle preferences help explain the persistent gender gap in academia? The “mothers work less” hypothesis supported for German but not for U.S. early career researchers
Source: PLoS One. 2018 Aug 28;13(8):e0202728. doi: 10.1371/journal.pone.0202728 (PMC6112653; doi:10.1371/journal.pone.0202728)

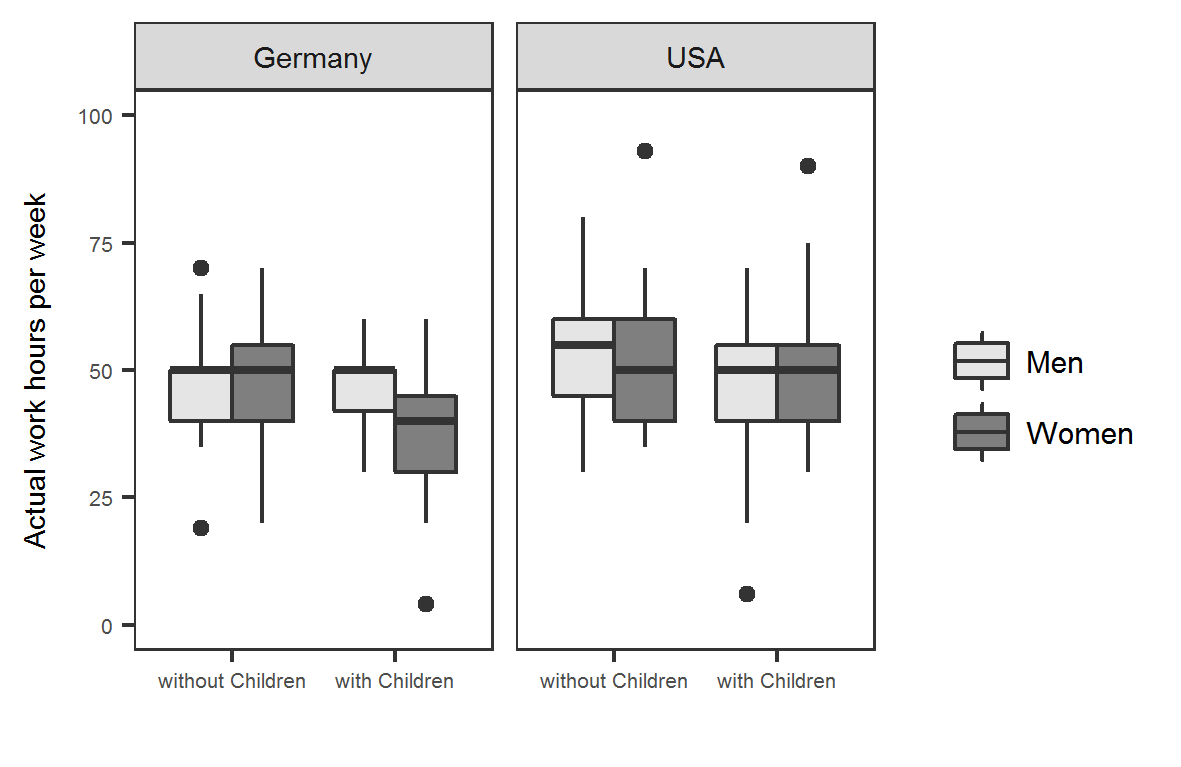

Supplement: S1 Fig — (TIFF) [file pone.0202728.s005.tiff]
